# Structure and Membrane Targeting of the PDZD7 Harmonin Homology Domain (HHD) Associated With Hearing Loss

**DOI:** 10.3389/fcell.2021.642666

**Published:** 2021-04-15

**Authors:** Lin Lin, Huang Wang, Decheng Ren, Yitian Xia, Guang He, Qing Lu

**Affiliations:** ^1^Key Laboratory for the Genetics of Developmental and Neuropsychiatric Disorders, Ministry of Education, Bio-X Institutes, Shanghai Jiao Tong University, Shanghai, China; ^2^State Key Laboratory of Molecular Biology, Shanghai Institute of Biochemistry and Cell Biology, Center for Excellence in Molecular Cell Science, Chinese Academy of Sciences, Shanghai, China; ^3^Key Laboratory for the Genetics of Developmental and Neuropsychiatric Disorders, Ministry of Education, Bio-X Institutes, Shanghai Jiao Tong University, Shanghai, China; ^4^Bio-X-Renji Hospital Research Center, School of Medicine, Renji Hospital, Shanghai Jiao Tong University, Shanghai, China

**Keywords:** usher syndrome, PDZD7, lipid binding, structure, deafness

## Abstract

Usher syndrome (USH) is the leading cause of hereditary hearing–vision loss in humans. PDZ domain-containing 7 (PDZD7) has been reported to be a modifier of and contributor to USH. PDZD7 co-localizes with USH2 proteins in the inner ear hair cells and is essential for ankle-link formation and stereocilia development. PDZD7 contains three PDZ domains and a low-complexity region between the last two PDZ domains, which has been overlooked in the previous studies. Here we characterized a well-folded harmonin homology domain (HHD) from the middle region and solved the PDZD7 HHD structure at the resolution of 1.49 Å. PDZD7 HHD adopts the same five-helix fold as other HHDs found in Harmonin and Whirlin; however, in PDZD7 HHD, a unique α1N helix occupies the canonical binding pocket, suggesting a distinct binding mode. Moreover, we found that the PDZD7 HHD domain can bind lipid and mediate the localization of PDZD7 to the plasma membrane in HEK293T cells. Intriguingly, a hearing-loss mutation at the N-terminal extension region of the HHD can disrupt the lipid-binding ability of PDZD7 HHD, suggesting that HHD-mediated membrane targeting is required for the hearing process. This structural and biochemical characterization of the PDZD7 HHD region provides mechanistic explanations for human deafness-causing mutations in PDZD7. Furthermore, this structure will also facilitate biochemical and functional studies of other HHDs.

## Introduction

Usher syndrome (USH) is the most common hereditary disease affecting both the visual and auditory systems in humans (Boughman et al., 1983; Keats and Corey, 1999; El-Amraoui and Petit, 2005). Based on the severity and progression of hearing loss, retinitis pigmentosa, and vestibular dysfunction, USH can be classified into three types: USH1, USH2, and USH3 (Gillespie and Müller, 2009; Friedman et al., 2011; Richardson et al., 2011; Pan and Zhang, 2012; Barr-Gillespie, 2015). USH2 is characterized by moderate-to-severe hearing impairment, progressive retinitis pigmentosa, and preserved vestibular function (Kimberling et al., 2010). Variants in the *USH2A*, *ADGRV1*, *WHRN*, and *PDZD7* genes are associated with USH2 in human (Weston et al., 2004; van Wijk et al., 2006; Ebermann et al., 2010). Stereocilia are actin-based protrusions on the apical surface of inner ear hair cells, essential for mechano-transduction in the cochlea. Stereocilia are organized in a staircase-like pattern and connected by several extracellular links, including tip link, ankle link, and basal link. USH2 proteins were reported to localize at the ankle link of hair cell stereocilia, but the molecular mechanisms governing ankle-link formation are not well characterized.

PDZ domain-containing 7 (PDZD7) is part of the ankle-link complex, playing a vital role in the development of cochlear hair cells (Grati et al., 2012; Chen et al., 2014; Hu et al., 2014; Zou et al., 2014; Du et al., 2020). *Pdzd7* knockout mice exhibit stereocilia disorganization, mechano-transduction reduction, and congenital deafness (Zou et al., 2014). At the molecular level, localization of USH2 proteins (USH2A, VLGR1, and Whirlin) at the ankle region is disrupted in *Pdzd7* knockout mice, suggesting that PDZD7 determines the localization of the USH2 complex. PDZD7 also interacts with USH1 proteins (USH1B, USH1C, and USH1G) (Schneider et al., 2009; Chen et al., 2014; Morgan et al., 2016). Mutations in PDZD7 are associated with non-syndromic hearing loss and contribute to digenic USH (Schneider et al., 2009; Ebermann et al., 2010). Recently, a novel mutation in *PDZD7*, p.525_533delDQERGRALLinsV was reported to be associated with non-syndromic hearing loss in the Chinese population (Wu et al., 2020).

The long isoform of PDZD7, which is indispensable for hair cell development (Du et al., 2020), contains tandem PDZ domains (PDZ1-2), an HHD (harmonin homology domain), a PR (proline-rich) region, and a third PDZ domain (PDZ3). The PDZ domains are responsible for interacting with USH2 proteins to form the ankle-link complex (Chen et al., 2014). However, the function of the PDZD7 HHD remains unknown. Due to the low similarity of the primary sequence, the HHD family was only recently classified and has been barely characterized (Faure et al., 2014). The first solved HHD structure was Harmonin HHD (Pan et al., 2009), with 80 amino acids folded into a compact five alpha-helix bundle. Interestingly, three hearing loss-related proteins (Harmonin, Whirlin, and PDZD7) contain this HHD, but the molecular mechanism of these HHDs is largely unknown. In previous studies of PDZD7, the region containing the HHD has been widely overlooked due to the low complexity of this region. Noticeably, the human deafness mutation mentioned above (p.525_533delDQERGRALLinsV in *PDZD7*) locates adjacent to the HHD region, implying its involvement with hearing loss.

In this study, we characterized the HHD region of PDZD7 and found it responsible for targeting PDZD7 to the plasma membrane. First, we designed two HHD constructs (a.a. 546–646 and a.a. 508–646), both of which form homogeneous and stable monomers in solution. Further, we solved the PDZD7 HHD structure at a resolution of 1.49 Å. PDZD7 HHD adopts a canonical five-helix fold and exists as a monomer. Intriguingly, PDZD7 has a unique extended helix at the N-terminus of the HHD domain (referred to as α1N), which is not observed in other HHD structures. α1N blocks the common pocket, which allows the Harmonin HHD to bind with SANS, suggesting a distinct structure feature of target recognition for PDZD7. Moreover, we discovered a lipid-binding property of the PDZD7 HHD domain *in vitro* and found that the HHD region is required for PDZD7 to localize to the plasma membrane in HEK293T cells. Furthermore, we found that a hearing-loss mutation of *PDZD7*, located in the N-terminal extension region of the HHD, can disrupt the lipid-binding ability of the PDZD7 HHD. Together with biochemical and structural characterization of the PDZD7 HHD region, we propose that PDZD7 HHD-mediated lipid binding is required for the hearing process. Our findings will also expand our knowledge on the action mode of HHDs.

## Materials and Methods

### Constructs, Protein Expression, and Purification

For PDZD7 (UniProtKB: E9Q9W7), HHD (HHD, a.a. 546–646) and HHD-Long (HHD-L, a.a. 508–646) were amplified by standard PCR (Vazyme, Nanjing, China) and inserted into pET.M.3C or pET.32M.3C through homologous recombination (Yeasen, Shanghai, China). For Whirlin, HHD2 (a.a. 410–510) was cloned into pET.M.3C. Mutants were created by PCR-based mutagenesis. All the plasmids were confirmed by DNA sequencing (GENEWIZ, Suzhou, China).

Proteins were expressed in BL21 (DE3) *E. coli* cells in LB medium at 16°C. Protein purifications were conducted using Ni^2+^-NTA affinity chromatography followed by size-exclusion chromatography (SEC) using a Superdex 200 column (GE Healthcare) in a buffer of 50 mM Tris–HCl, 1 mM DTT, 1 mM EDTA, pH 7.8, and 100 mM NaCl. Protein tags were cleaved using 3C protease and then removed by another step of size-exclusion chromatography.

### Crystallization, Data Collection, and Structure Determination

Crystals of PDZD7 HHD (∼10 mg/mL) were obtained at 16°C by the sitting-drop vapor diffusion method, in 0.1 M Bis–Tris pH 6.5 and 45% polypropylene glycol P400. Crystals were cryoprotected in reservoir solution with 20% glycerol. Diffraction data were collected at BL19U1 at Shanghai Synchrotron Radiation Facility (SSRF, Shanghai, China) and were processed with XDS.

The structure was solved by molecular replacement using the structure of Whirlin HHD2 (PDB code: 6FDD) as the search model by Phaser. Jelly-body refinement was performed with the obtained solutions using REFMAC in CCP4 (Murshudov et al., 2011), and the density maps were examined in Coot (Emsley and Cowtan, 2004). Further refinement was performed using Coot and REFMAC (Winn et al., 2011) iteratively. The final refinement statistics of the structure are listed in Table 1. Structural diagrams were prepared using PyMOL^1^.

**TABLE 1:** Data collection and refinement statistics.

**Data collection and processing**	
Crystal	PDZD7 HHD
Source	SSRF-BL19U1
Wavelength (Å)	0.97891
Space group	P2_1_
Unit cell (a, b, c, Å)	32.8, 75.0, 42.9
Unit cell (α, β, γ, °)	90, 103.1, 90
Resolution range (Å)	50–1.49 (1.55–1.49)
No. of unique reflections	32,592 (3,159)
Redundancy	6.7 (6.6)
I/σ (I)	13.5 (1.4)
Completeness (%)	99.5 (97.4)
R_*merge*_ (%)^*a*^	8.4 (13.5)
CC1/2	99.9 (71.1)
**Structure refinement**	
Resolution (Å)	37.50–1.49
R_*work*_^*b*^/R_*free*_^*c*^ (%)	17.71/19.40
rmsd bonds (Å)/angles (°)	0.013/1.280
**Number of reflections**	
Working set	30,582 (2,963)
Test set	1,998 (194)
Number of protein atoms	1,523
Number of solvent atoms	151
Average B factor (Å^2^) (Protein/solvent)	21.71/33.80
**Ramachandran plot (%)**	
Most favored regions	98.9
Additionally allowed	1.1
Outlier	0

*Numbers in parentheses represent the value for the highest-resolution shell.*

*^*a*^Rmerge = Σ| Ii–Im| /ΣIi, where Ii is the intensity of the measured reflection and Im is the mean intensity of all symmetry-related reflections.*

*^*b*^Rwork = Σ| | Fobs| –| Fcalc| | /Σ| Fobs|, where Fobs and Fcalc are observed and calculated structure factors.*

*^*c*^Rfree = ΣT| | Fobs| –| Fcalc| | /ΣT| Fobs|, where T is a test data set of about 5% of the total reflections randomly chosen and set aside prior to refinement.*

### SEC Coupled With Multi-Angle Static Light Scattering (SEC-MALS)

A pre-equilibrated Superose 12 10/300 GL column (GE Healthcare) was coupled to the AKTA FPLC system, a multi-angle static light scattering detector (miniDAWN, Wyatt), and a differential refractive index detector (Optilab, Wyatt). Protein samples were prepared for loading at 300 μl and 50 μM. Data were analyzed using ASTRA 6 (Wyatt).

### Circular Dichroism (CD)

Purified proteins cleaved from their tags were prepared. CD spectra were collected with a thermostated cell holder on a J-1500 spectropolarimeter (Jasco, Tokyo) at different temperatures (20–100°C). A quartz cell with a 1 cm path length was used for measurements in the far-UV region from 200 to 240 nm. The spectra of buffer alone were used to correct the sample spectra. Results were analyzed using Excel (Microsoft) and GraphPad Prism (GraphPad Software).

### Lipid-Binding Assay

A liposome stock was prepared by resuspending bovine brain lipid extracts (Folch fraction I, Sigma B1502) with HEPES buffer (20 mM HEPES, 100 mM NaCl, 1 mM DTT, pH 7.0). The stock at 5 mg/mL was dissolved by ultrasonication on ice. Protein samples at 0.3 mg/mL (without tags) were incubated with liposomes at room temperature for 15 min and then centrifuged at 100,000 × g for 40 min at 4°C in a Beckman TLA100.1 rotor. After centrifugation, the supernatants were collected to determine the unbound proteins. The pellets were washed and resuspended with loading buffer to determine the bound proteins. Both the supernatant and pellet proteins were subjected to sodium dodecyl sulfate-polyacrylamide gel electrophoresis (SDS-PAGE) and visualized by Coomassie blue staining.

### Cellular Localization

PDZD7-FL was cloned into pEGFP-C3, and the mutants ΔHHD-L, ΔHHD, Δ525–533, and L610DI613D were created. 293T cells were seeded into 12-well plates at the density of 2 × 10^5^ cells per well. The cells were cultured at 37°C in DMEM containing 10% fetal bovine serum (FBS) in 5% CO_2_. All the plasmids (3.3 μg each, the DNA concentration was in the range from 800 to 1,000 ng/μl) were transiently transfected into HEK293T cells via lipofection using the FuGENE HD kit (Promega, United States). After transfection, the cells were cultured for another 24 h before fixation. Cells were imaged with a TCS SP8 STED 3X Super-resolution Multiphoton Confocal Microscope (Leica, Germany). Fluorescence intensities of the plasma membrane and cytosolic regions were quantified by ImageJ software (National Institutes of Health, United States). Data were analyzed using unpaired *t*-test. Values (means ± SE) were calculated from three independent experiments (15 cells counted for each experiments).

## Results

### Structural Characterization of the PDZD7 HHD Domain

**FIGURE 1:**
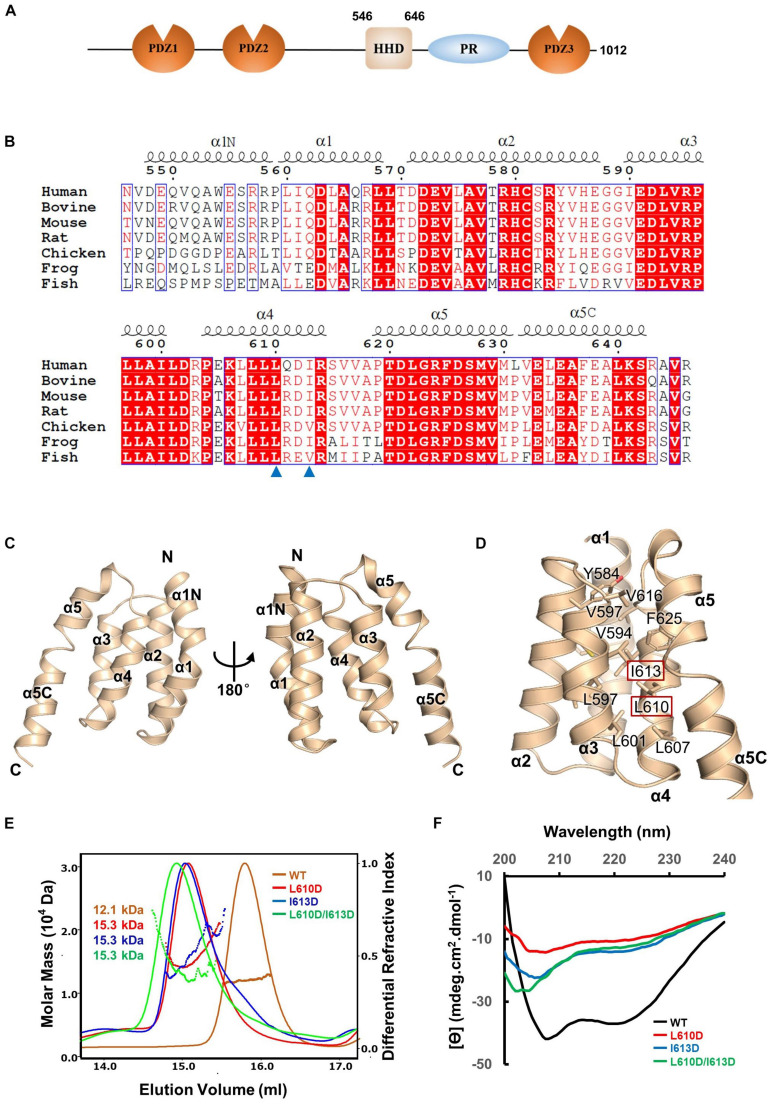
Crystal structure of the PDZD7 HHD domain. **(A)** Schematic diagrams showing the domain organization of PDZD7 long isoform. **(B)** Multiple-sequence alignment of PDZD7 HHD among different species. The secondary structural elements are indicated above the alignment. The identical residues are highlighted with red boxes, and the conserved residues are colored in red. Residues Leu610 and Ile613 are indicated by blue triangles. **(C)** Ribbon diagram representation of the PDZD7 HHD structure as viewed from the front (left) and the back (right). Secondary structure elements are labeled. **(D)** Residues involved in hydrophobic core formation are shown with the stick model. Residues Leu610 and Ile613 are highlighted with the red box. **(E)** FPLC coupled with static light scattering of wild-type HHD and its mutants, L610D, I613D, and L610D/I613D. **(F)** Circular dichroism spectrum of wild-type HHD, L610D, I613D, and L610D/I613D.

To elucidate the molecular mechanism of PDZD7 for ankle-link formation and stereocilia development, we carried out sequence analysis and found out a highly conserved helical region (HHD) between PDZ2 and PDZ3 (Figure 1A). Based on sequence alignment and secondary structure prediction, we designed two PDZD7 constructs containing the HHD domain (a.a. 546–646 and a.a. 508–646, namely, HHD and HHD-L, respectively). HHD-L contains an N-terminal extension region (a.a. 508–545), which is highly conserved among species (Figure 1B) but not found in Harmonin HHD or other HHDs.

To obtain structural information, we performed crystallization screening on HHD and HHD-L; however, only HHD successfully crystallized. HHD shows good homogeneity and exists as monomer in solution (Figure 1E). We then determined the HHD structure at 1.49-Å resolution. The HHD adopts a canonical five-helix fold (α1–α5) (Figure 1C). In the structure, α1–α5 are aligned anti-parallel to one another, making a five-helix bundle with multiple hydrophobic interactions inside the bundle (Figure 1D). Interestingly, both α1 and α5 helices turn bend, and the extended bend regions were named as α1N (a.a. 547–559) and α5C (a.a. 631–643). α1N and α5C flank the cleft of α2/α4 and α3/α4, respectively, to contribute to the hydrophobic core formation.

To further elucidate the effect of the hydrophobic-interaction network, we designed two single mutations (L610D and I613D at the α4 helix) and one double mutation (L610D/I613D) to destabilize the hydrophobic interactions in the HHD (Figure 1D). SEC-MALS was carried out to characterize the protein behavior of these mutants (Figure 1E). The experimental MW of wild-type (WT) PDZD7 (12.1 kDa) matches its theoretical MW of 11.4 kDa, confirming that the PDZD7 HHD is monomeric in solution. Compared with the WT protein, the peaks of the mutants shifted forward, suggesting that the homogeneity of the mutants was disturbed (Figure 1E). CD spectroscopy data also showed that these mutations disrupted protein folding (Figure 1F). Taking these results together, hydrophobic interactions inside the five-helix bundle are important for the folding of the PDZD7 HHD.

### PDZD7 HHD Contains Unique α1N and α5C Regions

**FIGURE 2:**
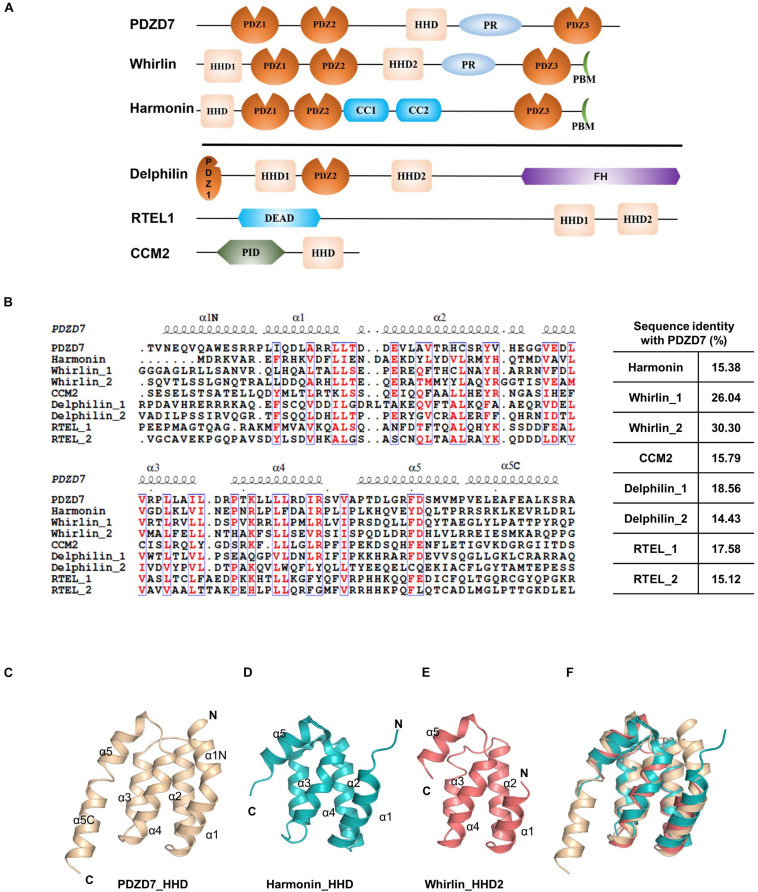
Conservation of HHD-containing proteins. **(A)** Schematic diagrams showing the domain organization of the six known HHD-containing proteins. **(B)** Sequence alignment of the nine known HHDs from *Mus musculus*. The conserved residues are colored in red. The sequence identity of other eight HHDs with PDZD7 is indicated. **(C–F)** Ribbon diagram representations of the PDZD7 HHD **(C)**, Harmonin HHD **(D)**, and Whirlin HHD2 **(E)** from the same view. **(F)** Structural superimposition of PDZD7, Harmonin, and Whirlin HHD2.

Until now, the HHD has only been identified in six proteins: PDZD7, Whirlin, Harmonin, Delphilin, regulator of telomere elongation helicase 1 (RTEL1), and cerebral cavernous malformation 2 (CCM2) (Figure 2A). Although these HHDs share a similar folding topology, the sequence similarities are very low and limited to residues forming the hydrophobic core (Figure 2B). Interestingly, three of the identified HHD-containing proteins are paralog USH scaffold proteins, namely, Harmonin, Whirlin, and PDZD7. Harmonin has an HHD at the N-terminal region coupled with its first PDZ domain, PDZ1, while Whirlin has one HHD (HHD1) in the N-terminal region and another HHD in the middle region(HHD2). PDZD7 HHD shares 26 and 30% sequence identity with HHD1 and HHD2 of Whirlin, respectively, and shares 15% identity with Harmonin. Superposition of PDZD7 HHD (Figure 2C) with Harmonin HHD (PDB: 2KBQ; Figure 2D) and Whirlin HHD2 (PDB: 6FDD; Figure 2E) indicates that they are well aligned and the overall folding is quite similar, with the RMSD on Cα of 1.81 and 1.37 Å, respectively. However, the extended regions, α1N and α5C, are not seen in Harmonin and Whirlin, suggesting that α1N and α5C are unique to PDZD7 (Figure 2F).

### PDZD7 HHD May Take a Distinct Target-Binding Mode

**FIGURE 3:**
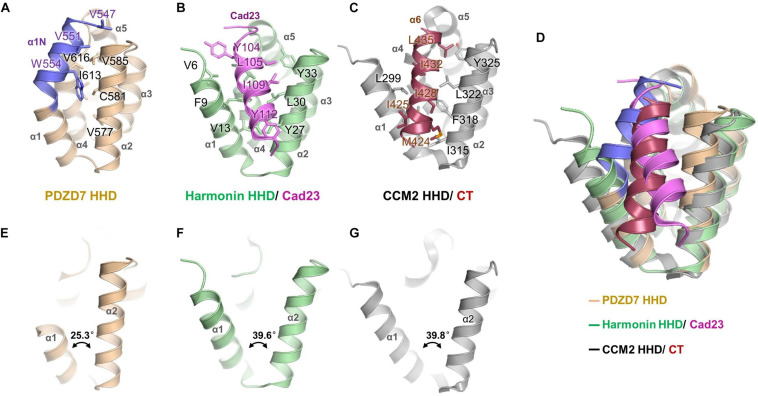
Structure comparison of PDZD7 HHD and other HHD complex structures. **(A)** Intramolecular interaction of PDZD7 HHD. The hydrophobic residues on the α1N helix and residues forming the α1/α2 cleft are labeled and shown as stick models. **(B)** Intermolecular interaction of Harmonin HHD and Cad23. The involved residues are labeled and shown as stick models. **(C)** Intramolecular interaction of CCM2 HHD and its C-terminal helix (CT). **(D)** Structural superimposition of PDZD7, Harmonin/Cad23, and CCM2/CT. **(E–G)** Structural scheme of the variability of α1/α2 cleft of PDZD7 **(E)**, Harmonin **(F)**, and CCM2 **(G)**.

A unique feature of the PDZD7 HHD structure is that the α1N helix turns back and contacts with the pocket formed between α1, α2, and α4. Val547, Val616, and Trp554 from α1N form extensive hydrophobic interactions with Val577, Cys581, and Val585 from α2, and Ile613 and Val616 from α4 (Figure 3A). Interestingly, this hydrophobic cleft between α1 and α2 is a common site in HHDs, such as in Harmonin and CCM2, and is required to recognize an isolated amphipathic helix on their binding targets.

In the typical target recognition mode of the HHD domain, such as in the structure of the Harmonin HHD/cadherin23 complex (PDB: 2KBR), the α1/α2 hairpin of Harmonin forms a hydrophobic cleft (composed of residues Val6, Phe9, and Val13 from α1, and Tyr27, Leu30, and Tyr33 from α2) to accommodate the cadherin23 helix (involving residues Tyr104, Leu105, Ile109, and Tyr112), resulting in a tight interaction between the two proteins (Figure 3B). Similarly, in the CCM2 HHD/CT structure (PDB: 4YKC), the isolated amphipathic C-helix of CCM2 (involving residues Met424, Ile425, Ile428, Ile432, and Leu435) packs with the groove formed between α1/α2 (involving residues Leu299, Met303, Ile315, Phe318, Leu322, and Tyr325) to form a hydrophobic interaction (Figure 3C).

Structural superimposition of the three structures of PDZD7, harmonin, and CCM2 shows that the PDZD7 HHD is well-aligned onto Harmonin and CCM2, and the α1/α2 cleft of the PDZD7 HHD domain is occupied by α1N and is not available for canonical target recognition (Figure 3D). Besides, a deviation of the α1/α2 V-shaped cleft was observed (Figures 3E–G). In the Harmonin/Cad23 and the CCM2/CT structures, the α1/α2 angle is about 39.6° and 39.8°, respectively. However, the α1/α2 angle in the PDZD7 HHD structure is 25.3°, suggesting that it adopts a more compact conformation. These unique structural features suggest that the PDZD7 HHD may take a distinct binding or regulation mode for target recognition. However, our crystal structure reveals a static view of PDZD7 HHD, so future work is needed to understand the conformational dynamics of the α1N helix in solution. Clearly, additional structures of the PDZD7 HHD in complex with its binding target will be helpful in understanding the underlying mechanism, although no binding targets of PDZD7 HHD have been reported yet. Our structure provides a starting point for dissecting the target recognition mechanism of PDZD7 HHD.

### The N-Terminal Extension Region of HHD Is Responsible for Lipid Binding

**FIGURE 4:**
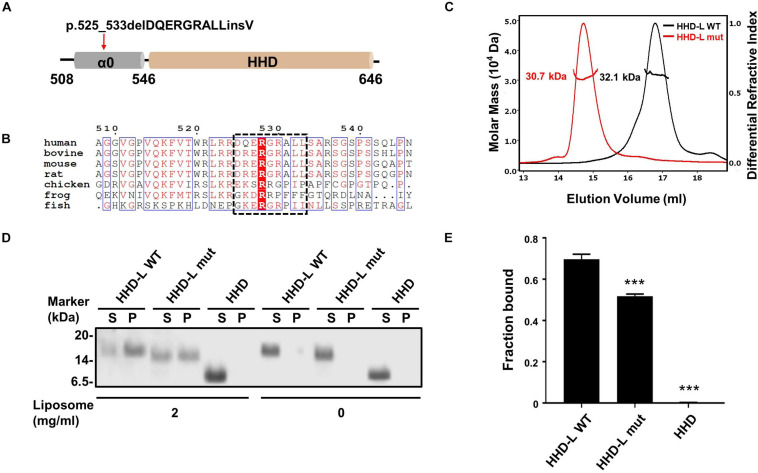
The N terminal extension region of HHD; α0 plays a role in lipid binding. **(A)** Schematic diagram of PDZD7 HHD-L (residues 508–646) to show the deafness-associated mutation, p.525_533delPQERGRALLinsV located at the α0 ahead of HHD. **(B)** Sequence alignment of the α0 region in different species. The deletion region in the deafness mutation is highlighted in black box. **(C)** FPLC coupled with static light scattering of two Trx-tagged proteins, wild-type HHD-L, and its mutation, Δ525–533 (HHD-L mut). **(D)** Co-sedimentation-based assay of tag-cut HHD-L WT, HHD-L mut, and HHD bindings to liposomes prepared from bovine brain lipid extract. After centrifugation, fractions labeled with S represent proteins in supernatants and fractions labeled with P represent proteins in pellets in the assays. **(E)** The bar graph represents the fraction of the protein recovered in the liposome-bound pellet in each sedimentation assay. Values are mean ± SD of three independent experiments. ***p < 0.001, using one-way ANOVA with Tukey’s multiple-comparison test.

*PDZD7* is a deafness-causing gene, and a previous report has associated PDZD7 mutations with USH, DFNB57, and autosomal recessive non-syndromic hearing loss (ARNSHL). Recently, a novel mutation in PDZD7, p.525_533delDQERGRALLinsV, has been shown to be associated with ARNSHL in the Chinese population. Notably, the eliminated nine residues (^525^DQERGRALL^533^) in this mutation are located adjacently to the N-terminus of the HHD (Figure 4A). Sequence alignment of the N-terminal extension region of the HHD (a.a. 508–545, named as α0 according to its secondary structure prediction) indicates that it contains a group of positively charged residues and is highly conserved among species (Figure 4B). To understand whether the deletion mutation affects the protein properties of the PDZD7 HHD, we performed SEC-MALS of WT Trx-tagged HHD^508–646^ (HHD-L WT) and Trx-tagged HHD^508–646^ Δ525–533 (HHD-L mut). The measured MWs of both HHD-L WT and HHD-L mut proteins (32.1 and 30.7 kDa, respectively) fit their theoretical MWs (30.0 and 29.1 kDa, respectively), indicating that they are monomeric in solution. Compared with HHD-L WT, the peak of HHD-L mut shifts forward, and the mutation affects the overall conformation of HHD-L mut and impairs the homogeneity of the protein (Figure 4C).

Considering that the N-terminal extension region of the HHD contains many conserved positively charged residues (Arg521, Arg523, Arg524, Arg526, Arg528, Arg530, and Arg536), we hypothesized that HHD-L might possess a putative lipid membrane-binding ability. We carried out a co-sedimentation-based liposome-binding assay to determine the lipid-binding ability of HHD-L WT, HHD-L mut, and HHD *in vitro*. Satisfyingly, approximately 72% of the purified HHD-L WT proteins bound to the liposome prepared from total bovine brain lipid extracts. In contrast, the liposome-bound pellet fraction of HHD-L mut decreased to ∼50%, possibly due to its loss of partial basic residues (Arg526, Arg528, and Arg530). Consistently, HHD, lacking the positively charged clusters, almost eliminated the lipid-binding capacity (Figures 4D,E). These results indicate that the N-terminal extension region is required for the lipid-binding ability of the PDZD7 HHD.

### HHD-L Is Essential for PDZD7 to Localize to the Plasma Membrane in HEK293T Cells

**FIGURE 5:**
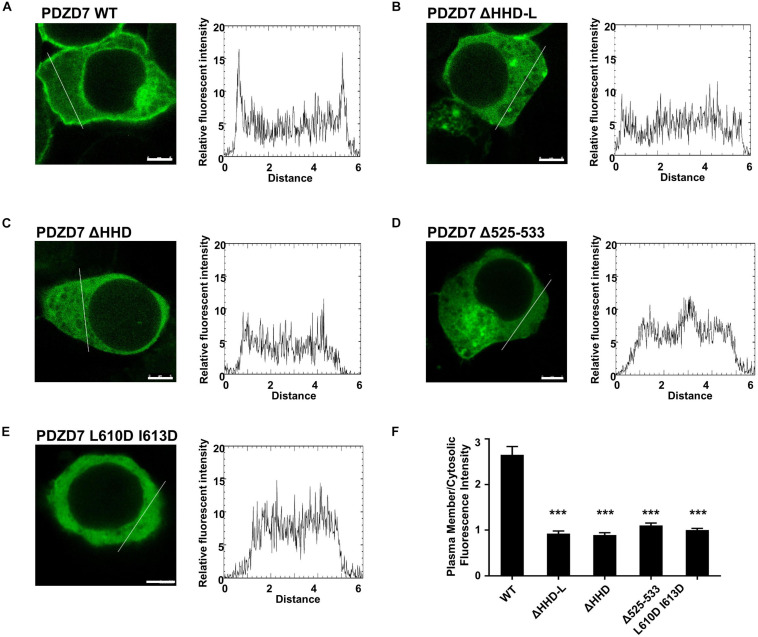
The subcellular localization of the PDZD7 FL wild type and mutants in HEK 293T cells. **(A–E)** Confocal image and elative fluorescence intensity in the membrane and cytosolic distributions of HEK293T cells transfected with GFP-tagged PDZD7 FL **(A)**, PDZD7 ΔHHD-L **(B)**, PDZD7 ΔHHD **(C)**, PDZD7 Δ525–533 **(D)**, and PDZD7 L610D I613D **(E)**, respectively. Scale bar: 5 μm. Fluorescence-intensity profiles represent the area marked by the white lines in **(A–E)**. **(F)** Quantitative analysis of membrane and cytosolic distributions of GFP-tagged PDZD7 FL, PDZD7 ΔHHD-L, PDZD7 ΔHHD, PDZD7 Δ525–533, and PDZD7 L610D I613D. Values are means ± SE from three independent experiments (15 cells per time), analyzed with unpaired *t*-test; ***p < 0.001.

Next, we assessed the lipid-binding properties of PDZD7 under cellular conditions. When overexpressed in HEK293T cells, some of the full-length PDZD7 localizes to the plasma membrane (Figure 5A), suggesting that PDZD7 possesses lipid membrane-binding ability in cells. To show whether membrane targeting of PDZD7 is mediated by the HHD-L region, we designed a construct named as PDZD7 ΔHHD-L by deleting the HHD-L region from the full-length PDZD7 sequence. As expected, deletion of HHD-L dramatically decreased the membrane localization of PDZD7 with most of PDZD7 diffused into the cytosol (Figure 5B), revealing that the HHD-L region plays an essential role in the membrane localization of PDZD7 in HEK293T cells. Intriguingly, ΔHHD alone also weakens the membrane localization of PDZD7 so that PDZD7 presents as a diffused distribution in the cytosol (Figure 5C), suggesting that the HHD domain and its N-terminal extension region are both required for facilitating PDZD7 membrane binding. Consistent with this hypothesis, substitution of two hydrophobic core residues in the HHD, Leu610 and Ile613 with Asp, also eliminates the membrane localization of PDZD7 (Figure 5E). These data suggest that the positive-charged N-terminal extension region and the core helical bundle may work together for membrane targeting.

More importantly, the hearing-loss mutation (Δ525–533) disrupts the plasma membrane-binding ability of PDZD7 (Figure 5D). This disease-causing mutation may affect the human hearing process by preventing PDZD7 from localizing to the membrane. It is also suggested that HHD-mediated membrane targeting is required for the hearing process.

To further verify if the membrane-targeting ability of PDZD7 is mediated by HHD-L, we overexpressed GFP-tagged PDZD7 HHD-L, HHD, HHD-L mut (Δ525–533), 1–507, and 508-end in 293T cells, respectively. As expected, a small part of HHD-L proteins localize at the plasma membrane, indicating its membrane association ability. HHD, HHD-L mut, and 508-end all present diffused distribution in cells, whereas 1–507 proteins form punctate enrichment in cytosol (Supplementary Figure 1). The results further confirmed that the N-terminal extension region is critical for the membrane targeting of PDZD7.

### Implications of the Hearing-Loss Mutations in the HHD Domain of Whirlin and PDZD7

The structural and biochemical characterization on the HHD domain can provide mechanistic explanations for human deafness mutations in both PDZD7 and Whirlin. As shown in Figures 4, 5D, the hearing-loss mutation at the α0 of PDZD7 HHD disrupts the lipid-binding ability of PDZD7 HHD *in vitro* and in HEK293T cells. Sequence analysis showed that two deafness-associated mutation sites in Whirlin (V460 and R490, NCBI Clinvar database, VCV000364688, and VCV000156029) are conserved between Whirlin and PDZD7, which are corresponding to V594 and R624 in PDZD7 (Supplementary Figures 2A,B). Interestingly, although these two sites are conserved, only mutants of Whirlin proteins present decreased homogeneity and stability (Supplementary Figures 2E,F) and PDZD7 mutant proteins have no impact (Supplementary Figures 2C,D). Our data suggest that these two disease mutations on Whirlin may disturb hearing by affecting the overall protein folding. Considering that three hearing-related proteins (Harmonin, Whirlin, and PDZD7) contain four HHD domains, our study not only provides mechanistic explanations to currently known disease-causing mutations, but also will be valuable in understanding functions of HHD domains in the hearing system.

## Discussion

PDZD7 is essential for ankle-link formation and stereocilia development; however, the structural and biochemical mechanisms of PDZD7 remain unclear. Here, we have characterized the HHD domain located in the middle region of PDZD7 and solved its crystal structure at high resolution. For the first time, we have reported the lipid-binding ability of PDZD7 in this study and found that the HHD region is responsible for targeting PDZD7 to the plasma membrane in HEK293T cells. Further, we have shown that a hearing-loss mutation in humans, located at the HHD N-terminal extension region, can disrupt the lipid-binding ability of the PDZ7 HHD, suggesting that the HHD-mediated membrane targeting is required for the hearing process. PDZD7 constitutes the ankle-link complex together with Whirlin, USH2A, and VLGR1 at the ankle region of stereocilia. Noticeably, localization of all three USH2 proteins at the ankle region is disrupted in *Pdzd7* knockout mice, suggesting that PDZD7 determines the localization of the USH2 proteins and is essential for organizing the ankle-link complex in developing cochlear hair cells. Our results indicate that HHD-mediated membrane targeting of PDZD7 may be important for regulating its interactions with membrane proteins USH2A and VLGR1. Further work is needed to verify this hypothesis.

The structure and lipid-binding property of the PDZD7 HHD reported in our work will also facilitate biochemical and functional studies of other HHD domains. As shown in the structure (Figures 1C, 2E), PDZD7 HHD adopts the same five-helix fold as the other HHDs, but the canonical binding pocket of PDZD7 is blocked by a unique α1N helix. We speculate that PDZD7 may adopt a distinct binding mode for membrane targeting via its HHD region. Both the HHD and its N-terminal extension region are required for membrane binding, suggesting that the extended α0 may couple with the core five-helix bundle (Figure 5). These findings expand the knowledge of the HHD family and imply diverse folding and binding capacity of helical domains.

## Data Availability Statement

The datasets presented in this study can be found in online repositories. The names of the repository/repositories and accession number(s) can be found below: http://www.wwpdb.org/, 7DE7.

## Author Contributions

LL, HW, and DR performed the experiments. LL, HW, DR, YX, GH, and QL analyzed the data. LL, GH, and QL designed the research. LL and QL drafted the manuscript. QL coordinated the project. All authors commented on the manuscript.

## Conflict of Interest

The authors declare that the research was conducted in the absence of any commercial or financial relationships that could be construed as a potential conflict of interest.

## Footnotes

1 http://www.pymol.org

## Supplementary Material

Supplementary Figure 1 The subcellular localization of different PDZD7 constructs in HEK 293T cells. **(A–E)** Confocal image and elative fluorescence intensity in membrane and cytosolic of HEK293T cells transfected with GFP-tagged PDZD7 HHD-L **(A)**, PDZD7 HHD **(B)**, PDZD7 HHD-L mut **(C)**, PDZD7 1–507 **(D)** and PDZD7 648-end **(E)**, respectively. Scale bar: 5 μm. Fluorescence-intensity profiles represent the area marked by the white lines in **(A–E)**.

Supplementary Figure 2 The effects of deafness-associated mutations on Whirlin and PDZD7 HHD proteins. **(A)** Sequence alignment of Whirlin and PDZD7 HHD domains from different species. The totally conserved residues are highlighted with red box, and conserved residues are colored in red. Two deafness-associated mutation sites in Whirlin, V460 and R490 (V594 and R624 in PDZD7 correspondingly) are annotated below as black triangles. **(B)** The combined ribbon and stick representations showing V594 and R624 in PDZD7 HHD structures. **(C)** FPLC coupled with static light scattering of PDZD7 HHD WT, V594, and R490H. **(D)** Circular dichroism spectrum of PDZD7 HHD WT, V594, and R490H at 208 nm. **(E)** FPLC coupled with static light scattering of Whirlin HHD WT, V460I, and R490H. **(F)** Circular dichroism spectrum of Whirlin HHD WT, V460I, and R490H at 208 nm.
