# Supplementary figures and images for: Structure and Membrane Targeting of the PDZD7 Harmonin Homology Domain (HHD) Associated With Hearing Loss
Source: Front Cell Dev Biol. 2021 Apr 15;9:642666. doi: 10.3389/fcell.2021.642666 (PMC8083959; doi:10.3389/fcell.2021.642666)

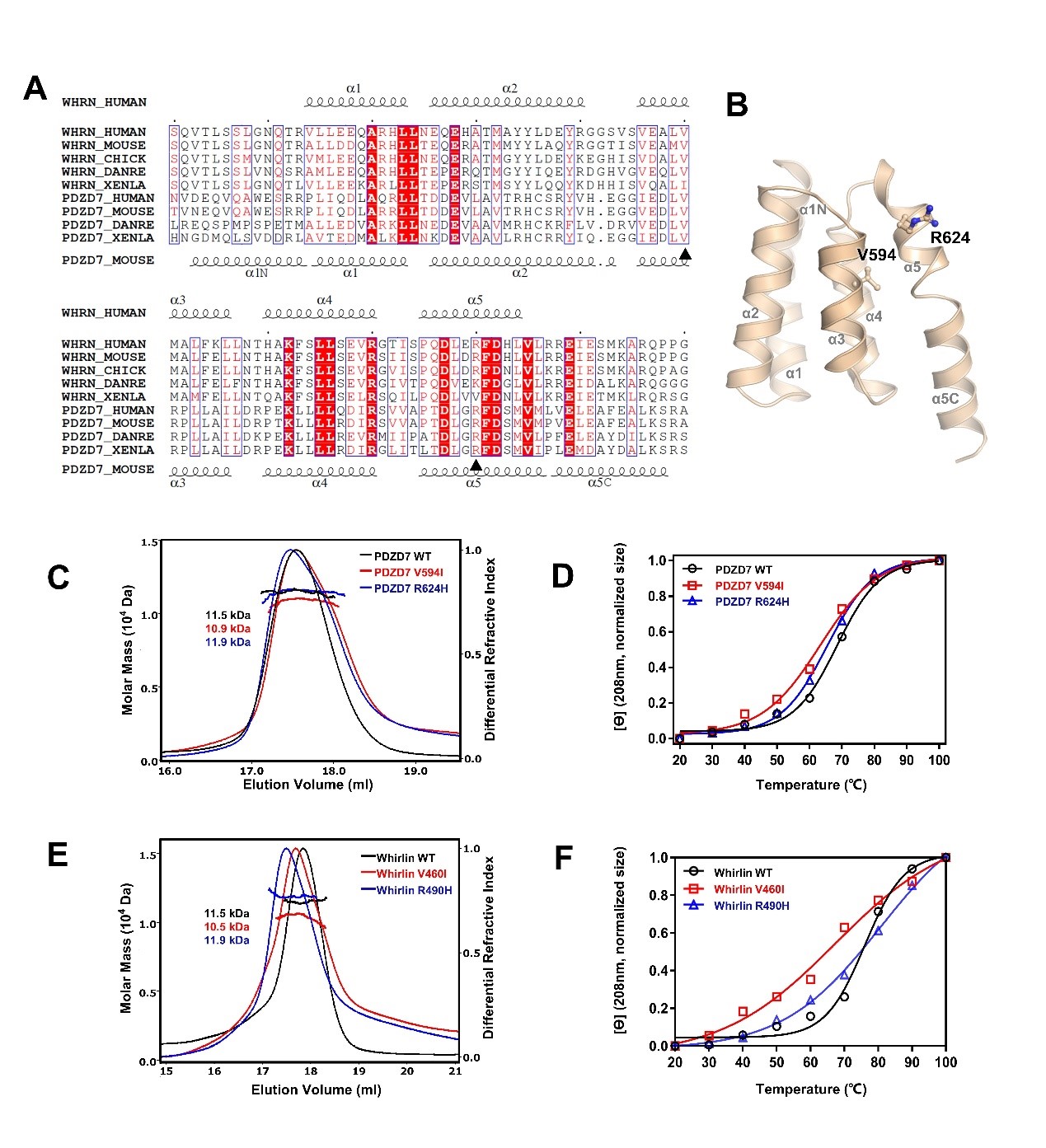

Supplement: Supplementary Figure 1 — The subcellular localization of different PDZD7 constructs in HEK 293T cells. (A–E) Confocal image and elative fluorescence intensity in membrane and cytosolic of HEK293T cells transfected with GFP-tagged PDZD7 HHD-L (A), PDZD7 HHD (B), PDZD7 HHD-L mut (C), PDZD7 1–507 (D) and PDZD7 648-end (E), respectively. Scale bar: 5 μm. Fluorescence-intensity profiles represent the area marked by the white lines in (A–E). [file Image_1.JPEG]

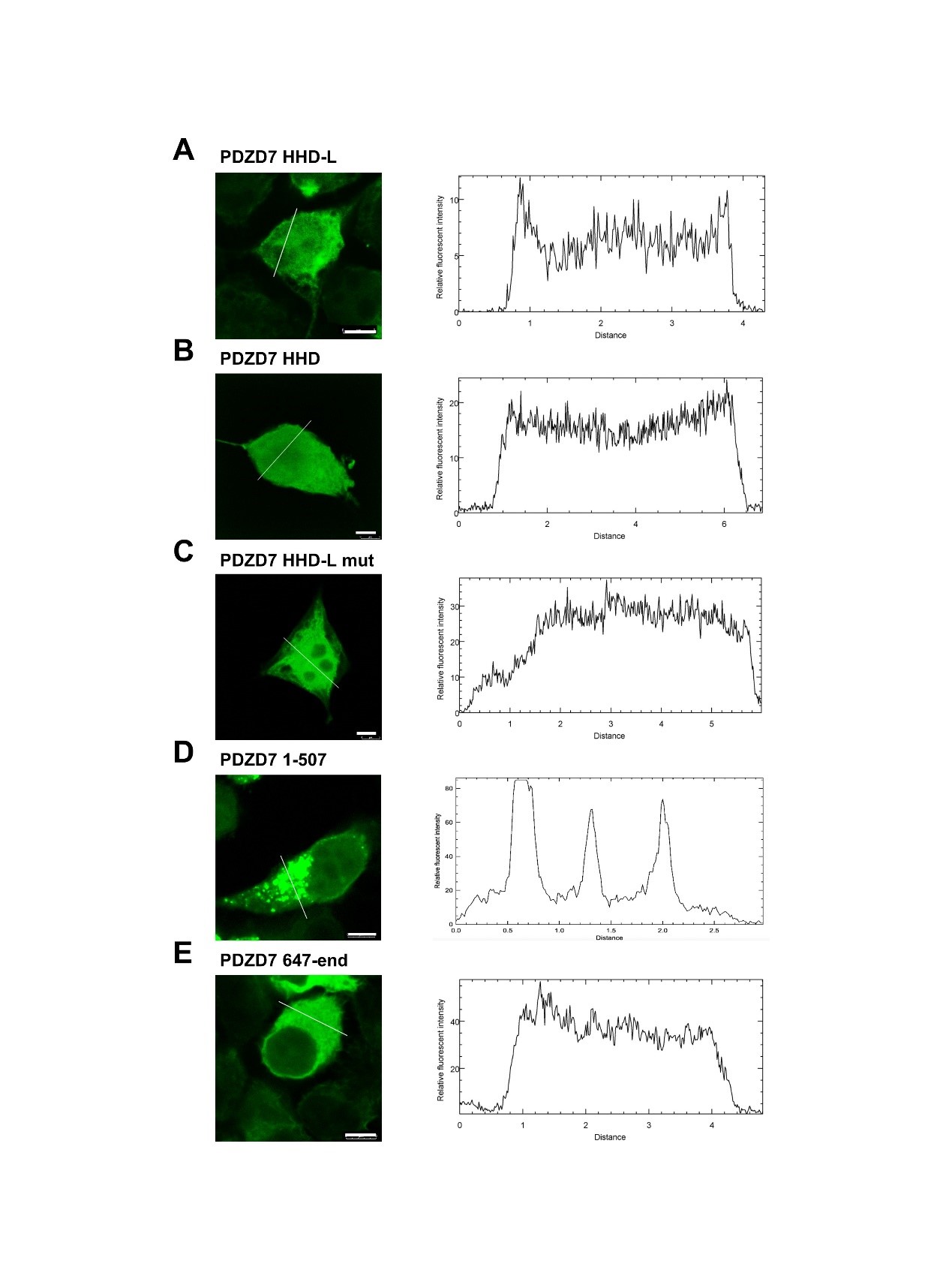

Supplement: Supplementary Figure 2 — The effects of deafness-associated mutations on Whirlin and PDZD7 HHD proteins. (A) Sequence alignment of Whirlin and PDZD7 HHD domains from different species. The totally conserved residues are highlighted with red box, and conserved residues are colored in red. Two deafness-associated mutation sites in Whirlin, V460 and R490 (V594 and R624 in PDZD7 correspondingly) are annotated below as black triangles. (B) The combined ribbon and stick representations showing V594 and R624 in PDZD7 HHD structures. (C) FPLC coupled with static light scattering of PDZD7 HHD WT, V594, and R490H. (D) Circular dichroism spectrum of PDZD7 HHD WT, V594, and R490H at 208 nm. (E) FPLC coupled with static light scattering of Whirlin HHD WT, V460I, and R490H. (F) Circular dichroism spectrum of Whirlin HHD WT, V460I, and R490H at 208 nm. [file Image_2.JPEG]
